# Computational Fluid Dynamics Modelling of Microfluidic Channel for Dielectrophoretic BioMEMS Application

**DOI:** 10.1155/2014/961301

**Published:** 2014-07-20

**Authors:** Wan Shi Low, Nahrizul Adib Kadri, Wan Abu Bakar bin Wan Abas

**Affiliations:** Department of Biomedical Engineering, University of Malaya, 50603 Kuala Lumpur, Malaysia

## Abstract

We propose a strategy for optimizing distribution of flow in a typical benchtop microfluidic chamber for dielectrophoretic application. It is aimed at encouraging uniform flow velocity along the whole analysis chamber in order to ensure DEP force is evenly applied to biological particle. Via the study, we have come up with a constructive strategy in improving the design of microfluidic channel which will greatly facilitate the use of DEP system in laboratory and primarily focus on the relationship between architecture and cell distribution, by resorting to the tubular structure of blood vessels. The design was validated by hydrodynamic flow simulation using COMSOL Multiphysics v4.2a software. Simulations show that the presence of 2-level bifurcation has developed portioning of volumetric flow which produced uniform flow across the channel. However, further bifurcation will reduce the volumetric flow rate, thus causing undesirable deposition of cell suspension around the chamber. Finally, an improvement of microfluidic design with rounded corner is proposed to encourage a uniform cell adhesion within the channel.

## 1. Introduction

The significance of biomedical application in miniatures system was realised since the introduction of microelectromechanical systems in the early of 1970s [1]. With an increase health care industry and awareness of microfluidic physic, biomedical microelectromechanical systems (BioMEMS) have turned out as an important subset of MEMS devices. Generally, BioMEMS refer to the system constructed using micron-and nanoscale fabrication technique that was used for analysis and delivery of biological and chemical particles. The device that integrated with this system is known as lab-on-a-chip and micrototal analysis system (TAS). Area of BioMEMS research and application included diagnostic, therapeutics, organ development [2], biomicroelectrode [3], tissue engineering [4], and bioinspired material for self-healing [5]. However, diagnostic application shows large researched segment presented in literature by many groups of study, where they are normally used to detect cells, proteins, microorganisms, viruses, and molecules of interest. For the purpose of rapid detection and cell characterization, microfluidics as powerful network of BioMEMS platforms is commonly used in diagnosis application.

In a laboratory, many different techniques are used in order to examine and analyse specimens. Microfluidics is the one of the technology of systems which is used to study fluid transport process, in channels with dimension of tens to hundreds of micrometres [3, 6]. The use of microfluidic channel has scaled down the fluidic processes to microscale with the advantage of smaller reagent volumes [7], shorter reaction times, and lower cost [8]. As the result of microfluidic research, lab-on-a-chip devices (LOC) are established to integrate laboratory functions into one chip for analytical process of biological and chemical samples [9, 10].

Separation of suspended micrometer seized particles is of fundamental importance in diagnostic microenvironment. One of the widely separation techniques used in BioMEMS diagnostic application is dielectrophoresis (DEP) [11, 12]. Dielectrophoresis (DEP) refers to a net force on the dielectric particles in response to a spatially nonuniform electric field. It is commonly proposed for physical manipulation and characterization of various types of cells and particles [13]. In literature, DEP has been employed to separate live and dead cells [14], nonferrous particles [15], DNA molecules [16], and viruses [10, 17]. The expression for DEP force can be represented by

(1) 〈FDEP〉=2πε0εmr3Re[K(ω)]∇E2,

where *ε*_0_ is the free space permittivity, *ε*_*m*_ is the permittivity of medium surrounding the particle, *r* is the particle radius, *K*(*ω*) is the complex Clausius-Mossotti factor, and ∇*E* is the magnitude gradient of electric field, expressed in RMS value. As this technique applies forces on microscale, it is able to integrate seamlessly with microfluidics. However, there is a unique reliability problem for its flow measurement within microfluidic channel in these systems, where imprecision in the geometry of microchannel will cause variation in performance, such as nonuniform flow distribution [11].

In an idealized DEP configuration, the gradient of electric field emitted by microelectrode is assumed to be invariant of position and have constant value [18, 19]. Such a constancy of electric field gradient received by cell particle is crucial to induce efficient separation of particles based on their dielectric field properties. To do so, it is important to ensure a well distribution of particle above the DEP microelectrode device. The higher number of cell deposited around an area, the higher the DEP force generated to increase the sensitivity of DEP force received by each cell. However, it must be noted that cell distribution within microfluidic channel is highly influenced by flow velocity within microfluidic channel which is controlled by hydrodynamic drag force [20]. Tan et al. [21] have stated that the combination of motive electric field and the uniform fluid flow is important for continuous cell separation with DEP techniques, where the force experienced by particles within the fluid will influence its motion. It is further evidenced when suspension of cell is found within microfluidic channel due to the nonuniformity of fluid velocity field. Such a condition had developed uneven light intensity during cell collections analysis, thus influencing the DEP spectrum result [22]. Henceforth, to develop an accurate separation and characterization DEP technique, the design of microfluidic channel which encourages uniform velocity flow within it will provide great convenience to control the evenly deposition cell above the microelectrode devices.

### 1.1. Geometrical Design

In most cases, there are two types of systems used in microfluidic channels which are single phase flow and multiphase flow [4]. Single phase microfluidic system manipulates one phase flow, where the fluid does not change state during heating or cooling while multiphase flow displays numerous pattern behaviour, such as droplets [23], bubbles, slugs [24], or thin films [25]. Although multiphase based system governed majority of the microfluidic channel design, single phase microfluidic system shows high suitability for DEP application due to its ability to control pressure drop within channel [26]. Aforementioned, DEP technique has high potential in cell separation due to its ability to differentiate their dielectric differences. By adapting single phase microfluidic system into DEP application, it is believed that the yield can be further increased by utilizing a good channel design to characterising cell.

Under such a system, the flow distribution generated within different cross section of microfluidic channel had been purposed and studied since 1965, such as single long uniform circular section channel [27], rectangular channel [28], and hyperelliptical cross section microfluidic channel [29]. Among all, microfluidic channel with rectangular cross section predominantly gained high interest in literature due to its ease of fabrication and predictable flow. Apart from cross section, for DEP application, the presence of chamber in the centre of microfluidic channel is important to help localise the cell at microelectrode. In study done by Saias et al. [19], a diamond shape of microfluidic channel has been proposed to develop uniform flow within it. Such a geometrical design has adjusted the hydraulic resistance and thus improved the uniformity field within the microfluidic channel. Although its results meet the aim of our study objective, the narrows walls with high level of branches will cause difficulty during microfabrication process as it required high technology and it is hard to be achieved with equipment available in our laboratory. Furthermore, practically, the sharp corner of such a geometrical design will increase the contact area and endure larger shear stress during fluid flow. Henceforth, it will result net force drags and cells might accumulate around the corner and thus affect the quantity of cells to be trapped in the cell chamber [30], such as single long uniform circular section channel, rectangular channel [28], and hyperelliptical cross section microfluidic channel [29]. Among all, microfluidic channel with rectangular cross section predominantly gained high interest in literature due to its ease of fabrication and predictable flow.

In context of this study, improvement will be done based on typical benchtop-fabricated microfluidic channel design, previously used in study by Fatoyinbo et al. [22]. While evaluating the velocity field generated in each design quantitatively, a mathematical model based on the numerical resolution of Navier-Stokes equation has been developed with COMSOL Multiphysics v4.2a to simulate the flow in microchannel for non-Newtonian fluids. In the end of this paper, flow pattern and fluid velocity for different geometrical designs are obtained and the comparison between different geometrical designs is made. This paper will also outline the best geometrical design in order to produce consistent microfluidic flow within the channel.

## 2. Proposed Strategy

### 2.1. Requirements

In proposing a geometrical design of microfluidic channels, the first step is to define the required specifications. In order to successfully develop a microfluidic channel that meets the design need, several parameters are taken into careful consideration, including sample size, developed channel flow pattern, optimum fluid mean velocity, uniformity of flow, and fabrication complexity as described in the following.

#### 2.1.1. Sample Volume

Sample volume plays an important role while designing a microfluidic channel. The relationship between sample volume (*V*) and analysed concentration is shown below [9]:

(2) $$\begin{array}{c} V=\frac{1}{\eta_{S}N_{A}A_{i}}, \end{array}$$

where *η*_*S*_ indicates the sensor efficiency, *N*_*A*_ is* Avogadro's* number, and *A*_*i*_ is the analyte concentration. Although microfluidic channel enables the use of smaller reagent for the analysis purpose, the effect of reduced volumes will influence the number of analysed targets available in the study. Therefore, it is indeed necessary to determine the amount of sample required to perform an analysis given the level of detector ability [9]. In order to capture the optimal volume of microfluidic channel without influencing the velocity flow, the length and width of the microfluidic chamber are suggested to be 7 mm and 5 mm, respectively.

#### 2.1.2. Optimum Fluid Mean Velocity

When the specimen is pushed into the chamber, the force applied will influence the flow velocity of microfluidic channel. When the flow velocity is increased, the flow rate will be increased as well. However, higher flow velocity will cause the changes in fluid properties such as the amount of cells in accordance with location. Hence, the flow of velocity parameter should be adapted in a certain range. In cell capture application for DEP system, the fluid mean velocity should approximate to 0.003 ms^−1^ to allow the sufficient cell capture within the chamber [4, 9]. To reach the optimum flow rate, the length of the entire microfluidic channel should fall within the range from 23 to 30 mm.

#### 2.1.3. Fabrication Complexity

Although microfabrication offers immense toolbox to process and fabricate the gasket, the complexity of microfluidic system can result in higher cost because different channels depths and dimensions require a series of fabrication procedures which will involve sophisticated technologies. In this case, it is important that the proposed geometry is able to industrialise to DEP use devices as well as develop uniform deposition of cell within it.

The microfluidic channel with rectangular cross section is selected in our design due to the ease of fabrication. For such a microfluidic channel, the flow would be completely laminar if Reynolds number is less than 200 [30]. Reynolds number is defined as the ratio of inertia forces to viscous forces:

(3) Re=inertia  forceviscous  force=ρVDμ,

where *ρ* is the fluid density, *V* is the mean fluid velocity, and *D* is the diameter of microfluidic channel while *μ* is the fluid viscosity. Since water will be used as the main material flow through the channel during the simulation process, thus fluid viscosity will remain the same for all proposed geometries. In order to achieve laminar flow, the inlet diameter of microfluidic channel should be kept as small as possible, with the maximum value of 1 mm in our study. Besides, the depth of the microfluidic channel needs to be constant across the whole microdevice. Due to the constraint of available microfabrication technique, the depth of channel has been fixed to 1 mm.

#### 2.1.4. Uniformity of Flow

Finally, in our application, it is necessary to make sure that the distribution of cell suspension is as uniform as possible throughout the microfluidic channel. Aforementioned, any sedimentation of cell within microfluidic channel can exert influential impact on the DEP analysis. Thus, to ensure uniform loading capacity for a given chamber size, it is crucial that the whole area reaches saturation at the same time. In this case, cross section of microfluidic channel should be increased and it will be further discussed later.

### 2.2. Network Architecture

**Figure 1:**
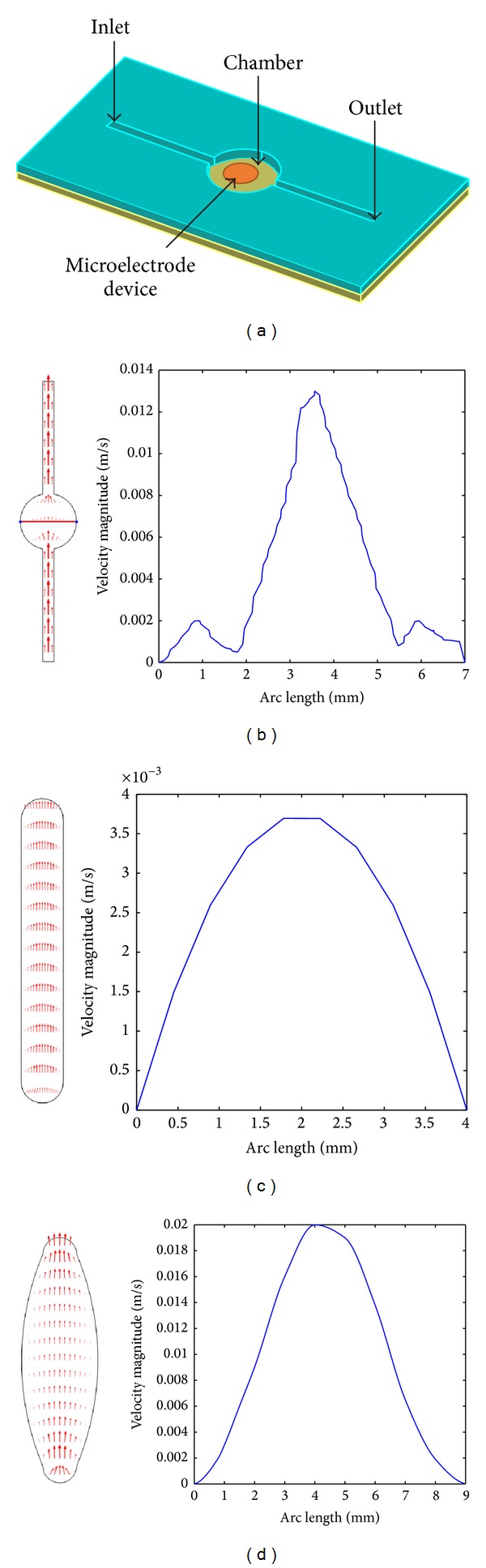
A typical benchtop-fabricated microfluidic channel's (a) components with various gasket design velocity profile as shown in (b), (c), and (d). Arc length refers to the channel length in the middle axis as represented by the red line in (b).

Generally, a typical benchtop-fabricated microfluidic design consists of three important parts, which are an inlet, an outlet, and a chamber as illustrated in Figure 1(a). In this system, the gasket of microfluidic channel is attached above electrode layer, to create a chamber for DEP experiment to take place. Cell suspension is inserted into the inlet and deposited around the microelectrode device where the actual effect of DEP is applied. However, as syringe pump is the controller used in most microfluidic, it will develop Poiseuille flow within microfluidic channel. Such a flow causes maximum velocity along the mean line of the middle microfluidic channel, as illustrated in Figure 1(b). Furthermore, the simulation had showed 2 major constraints in this simple architecture. Firstly, the maximum flow rate was 0.013 ms^−1^ in contrast to the maximum theoretical rate, which is 0.003 ms^−1^. Therefore, it will never reach the maximum analysis throughput. Secondly, as shown in Figure 1(b), such a system does not have the same velocity flow across the chamber. Although another 2 different channel designs (see Figures 1(c) and 1(d)) were proposed in the study to help improve the cell distribution, both designs depicted parabola velocity field. As akin to Figure 1(b) design, these geometrical designs will lead to the inhomogeneous cell accumulation in the chamber, which is undesirable in our study. Therefore, in order to optimize the loading capacity with uniform cell distribution around the chamber, it is necessary to modify the channels and chamber architecture to flatten the velocity dispersion.

**Figure 2:**
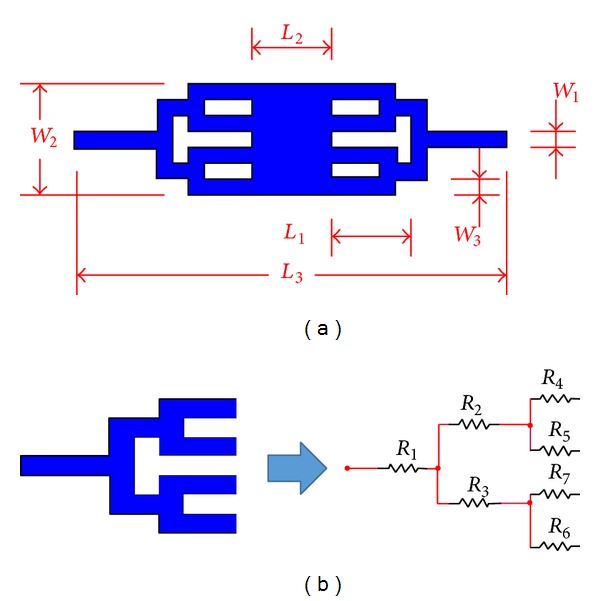
Schematic design of (a) tree-like flow division microfluidic channel with its (b) equivalent electric circuit analogy.

To solve the constraints for DEP microfluidic systems, a microfluidic geometry that split the flow into multiple channels as illustrated in Figure 2(a) is proposed in this study. The inspiration of such a design is from the tubular network of blood vessels. The flows from the inlet will flow toward the bifurcation in two outlet branches, in which the downstream branches are symmetric with respect to the inlet. Flows from two branches of downstream bifurcation will designate into another 4 subchannels. The fluid flow will be combined into a single stream at the outlet.

By mimicking the vascular hierarchical structure, the presence of multiple stage division in this design is able to generate hydraulic resistance within each daughter channel, thereby improving the flow control through the microfluidic channel. In contrast to biological vascular network in which its channel cross-sectional surface is getting smaller for each bifurcation, our proposed design is restricted to channels with a similar cross section in order to fulfil the laminar flow requirements. Furthermore, it can help preventing pressure build-up in the microfluidic channel which may lead to deformation.

### 2.3. Network Characterization

To produce a particular output response at the drain outlet ports, thoughtful consideration is needed to determine the cross-sectional dimension of the channel. The specification of total length (*L*_3_) and the width of inlet port (*W*_1_) for our design have been defined before, where the total length of microfluidic channel will be set within the range from 23 mm to 30 mm. Meanwhile the inlet port (*W*_1_) and the depth of the microfluidic channel were fixed to 1 mm due to the limitation of fabrication technique available in our lab.

As mentioned beforehand, cell will deposit around chamber where DEP effect takes place. Therefore, the dimension of chamber must be big enough to locate each suspension of cell while generating a uniform flow against it. According to Saliterman [9], the cell suspension is optimum at the length of chamber (*L*_2_) with 7 mm. In the meantime, the total width of the chamber (*W*_2_) is dependent on the cross section of each drain outlet port, which can be further determined by applying Murray's Law and electric circuit analogy.

Based on Murray's Law, the cross section and shape of drain channel at *n*th level must be the same [31] in order to equalise the hydraulic resistance of each part and thereby generate a uniform flow distribution within the whole wide chamber. Hydraulic resistance is purely related to the drain channel length [3]. It can be evidenced by considering the flow of a fluid within the channel behave akin to the flow of electron in electric circuit (Figure 2(b)). The hydraulic resistance generated within each channel acts as a medium to slow down the fluid flow within it, thus evenly distributing fluid particles across the chamber at a constant velocity.

Apart from hydraulic resistance, the length of channel at *n*th level too plays an important role in determining the compactness of the overall design. By referring to Figure 2, the compactness parameter [19] is equal to

(4) C=L1W2.

The lower the calculated *C* value, the higher the compactness level of the microfluidic geometric. For laboratory DEP application, the calculated compactness should fall in between 0 and 1 for a microfluidic design.

### 2.4. Improvement of Design and Comparison

In order to fulfil the requirements of our design as mentioned earlier in this section, a series of architectures which give raise from tree-like flow division microfluidic should be tested. The length of *n*th channel is the key determinant of the flow and the compactness of the design. To enhance the compatible compactness of the design while preserving uniform flow distribution, the velocity profile generated by different length fractions between mother and daughter branch will be too compared.

Furthermore, Emerson et al. point out that the volumetric flow rate is halved at each bifurcation and the relationship between the mean velocities of *n*-folded network can be written as [31]:

(5) VnV0=2−nA0An.

This statement has become the field of interest in our study to further investigate the relationship between levels of bifurcation with the velocity profile. A 3-level of bifurcation microfluidic channel will be compared with our proposed 2-level of bifurcation microfluidic channel.

## 3. Materials and Methods

### 3.1. Computational Fluid Modelling Method

To quantitatively evaluate the uniformity of flow and velocity field in the middle of the analysis area, the fluid flow within the proposed microfluidic channel was simulated with COMSOL Multiphysics 4.2a (COMSOL Inc., Palo Alto, USA), with primary focus on the module such as laminar flow and particle tracing for fluid flow. Three assumptions were made to mimic the actual flow situation, included

1. the fluid is Newtonian,
2. no-slip boundary condition,
3. the flow within the microfluidic channel is incompressible.

To generate 2D model, the microfluidic architecture was first created by using AUTOCAD 2011 (Autodesk Inc., USA). The design geometry would then be imported into COMSOL model library. Simulation was performed using the steady state Navier-Stokes model, where the fluid inside the channels was simulated as water (Newtonian fluid). The model particles are chosen so as to have dynamic viscosity of yeast cells suspended in an aqueous solution. The dimension of the yeast cells was set to range from 4 *μ*m to 6 *μ*m. The inlet and outlet ports were specified at the beginning and end of geometry. Meanwhile, the inlet velocity flow was adjusted accordingly to the channel length (e.g., if the length of entire microfluidic channel is 23 mm, the inlet velocity flow is 0.023 ms^−1^). The channel fluid flow study was computed and model surface plot which showed the velocity magnitude would be generated. In order to study the fluid distribution within the chamber, the velocity field fluctuations in the middle of chamber were analysed.

## 4. Result and Discussion

### 4.1. Proposed Microfluidic Architecture

**Figure 3:**
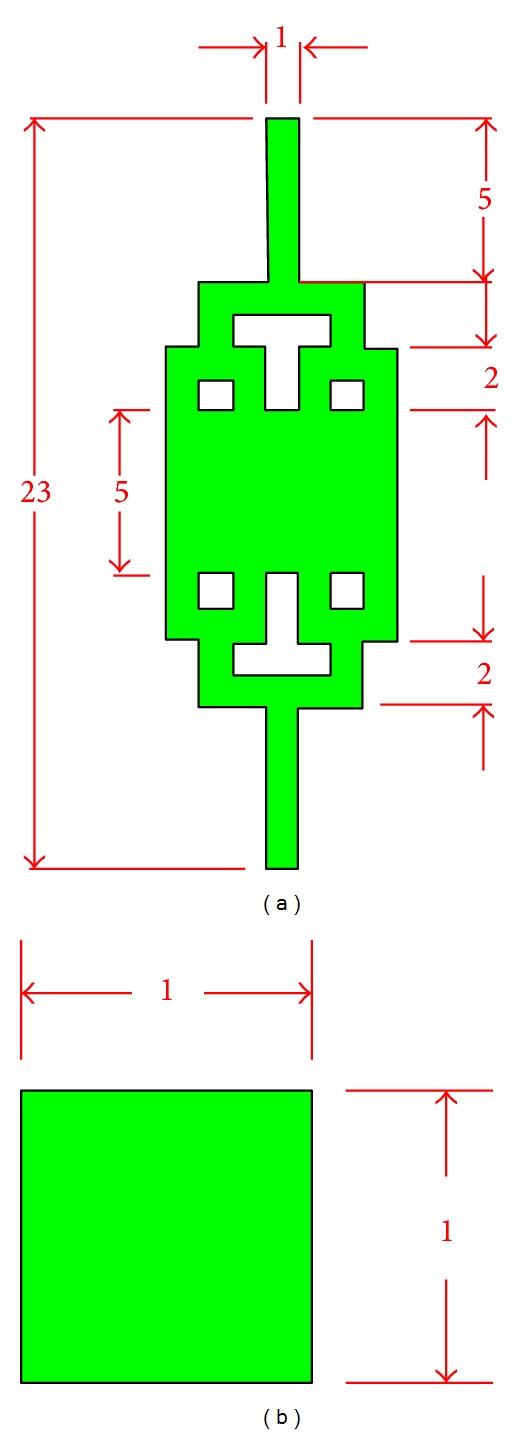
Schematic of (a) the proposed microfluidic channel architecture with (b) its channel's cross-sectional dimension.

A schematic of proposed tree-like microfluidic channel geometrical design is plotted with AutoCAD 2011 as illustrated in Figure 3. All dimensions used are in millimetres. For this proposed microfluidic design, we will consider rectangular cross section with reactive hydrophilic walls microchannel. The width of the inlet channel, *W*_1_, and the length of entire microfluidic channel are fixed to 1 mm and 23 mm, respectively in order to obtain a Reynolds number smaller than 200. As aforesaid, the compactness should not exceed 1 for DEP application in our design. In this context, microfluidic channel which is extremely long will be impractical as the increase in length will increase the space usage in return. In this design, our parameter of compactness, *C*, is 0.42 and, thus, it fulfils the design requirement.

**Figure 4:**
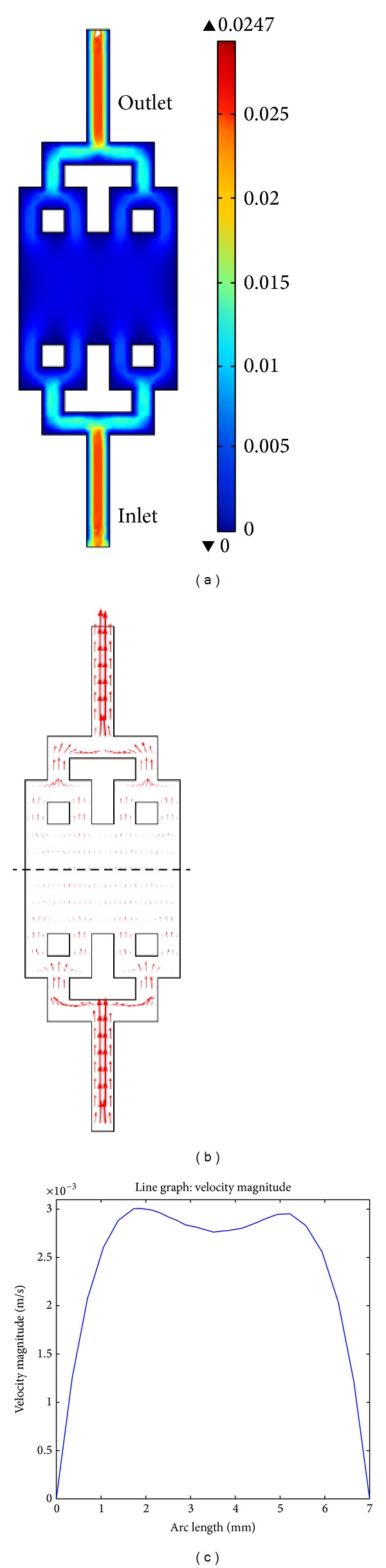
Fluid flow analysis of (a) velocity field surface plot. The colour spectrum bar shows the velocity field generated in the fluid system; (b) surface arrow plot and (c) the average velocity across the length of the middle microfluidic channel (represented by dashed line).

When the fluid is drawn into the channel, the Reynolds number can be divided into three different zones, which are the entry zone, the Poiseuille zone, and the surface traction zone. The entry zone is the first to form near the inlet and is characterized by high and time dependent velocities. The second region is the Poiseuille zone, which has the classical fully developed parabolic profile and it is ended with a traction region. At traction region, the 2-level bifurcation geometrical design will flatten the high velocity field generated in Poiseuille zone. It can be further proven with our COMSOL 2D simulation results [32]. The 2D simulation results are presented in 3 forms: surface plots of velocity field (Figure 4(a)), surface arrow plot (Figure 4(b)), and line graph of velocity magnitude (Figure 4(c)). The surface velocity plot (Figure 4(a)) indicates the velocity field within proposed microfluidic channel geometry in colour spectrum. When the cell suspension is pushed into inlet, the velocity around the inlet region is approximating to 0.0247 mm^−1^ (represented by red region). The presence of microfluidic division network in our design is proved to be slower down the fluid stream velocity by dividing the single volumetric flow into multiple channels. The high velocity profile at the inlet channel will be flattened down by the first bifurcation. Meanwhile the nonuniform particle profile in the first bifurcation is then removed by the second bifurcation. Due to equalization of hydraulic resistance in each channel, this proposed flow distribution network has enhanced a uniform distribution of flow across the microfluidic chamber, where an evenly blue spectrum is obtained around the microfluidic chamber with COMSOL simulation. Such a condition can be further proved by surface arrow plot (Figure 4(b)). The length of the arrows in this plot is representative of the average flow velocity. Since the tiny arrows across the chamber have similar length, thus this indicates the uniform velocity field around the chamber.

In order to quantitatively evaluate the velocity field fluctuation in the middle of the chamber, the graph of average velocity across the middle arc length is plotted (Figure 4(c)). As compared with the typical benchtop microchannel design velocity measurement depicted in Figure 1, the maximum fluid velocity obtained from the proposed design is 0.003 ms^−1^, thus fulfilling the requirement, where the fluid mean velocity should approximate and not exceed 0.003 ms^−1^ in order to allow sufficient cell capture within the chamber for DEP analysis. Besides, such a tree-like flow division microfluidic channel (Figure 3) has showed better velocity distribution within the microfluidic chamber in contrast with previous microfluidic design (Figure 1). Unlike the previous velocity profile (Figure 1(b)) which showed a steep increase and decrease in velocity between 2 and 5 mm, the quadratic-shaped velocity profile of our proposed design indicates stable uniform flow within the channel. Therefore, the localised build-up of cells over times due to the low velocity of fluid flow can be reduced in our design.

Although this proposed design has shown some improvement, the velocity profile within distance of 2 mm from the wall showed low velocity field. Such a condition is caused by the generation of shear stress which acting on the wall surface in the direction of fluid flow. Besides, the velocity curve was found to be asymmetric within a symmetric model, where the left peak value was slightly higher than the right peak value (see Figure 4(b)). It is due to the selection of yeast cells dimension such as size and density. Aforementioned, the size of yeast cells in this numerical study was varied within the range from 4 to 6 *μ*m in order to fit the model as close as to the experimental work. As a consequence, the cells will travel in with different velocity according to their mass and size, thus resulting in asymmetric velocity graph.

### 4.2. Effect of Length Fraction of Subchannels

**Figure 5:**
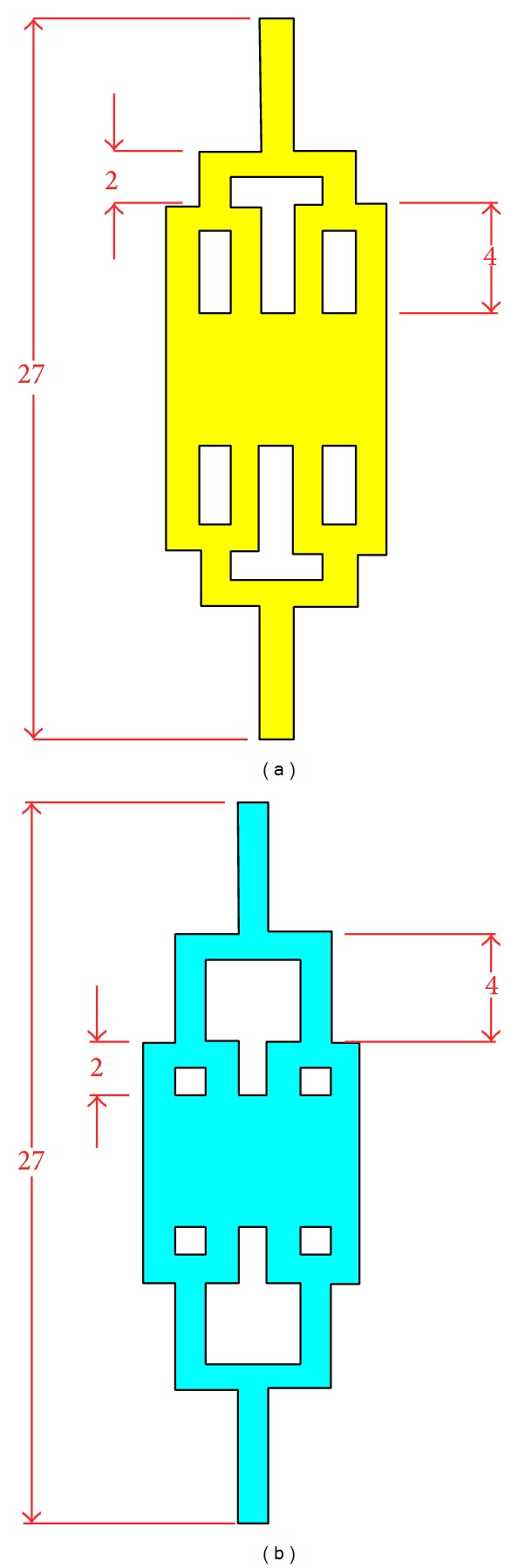
Schematic of (a) microfluidic platform architecture with subchannel length ratio of 1 : 2; (b) microfluidic with subchannel length ratio of 2 : 1.

As described in network characterization, the length of subchannel from one to the next plays an important role in identifying the flow and the compactness of the design. To investigate the relationship between subchannel length fraction and the velocity field across the channel, the simulation based on the geometrical design as illustrated in Figure 5 is generated. The geometrical parameter such as channel width, length of inlet channel, and length of chamber remains the same as our original proposed design. The compactness for both geometries is 0.714 which fulfils the design requirement.

**Figure 6:**
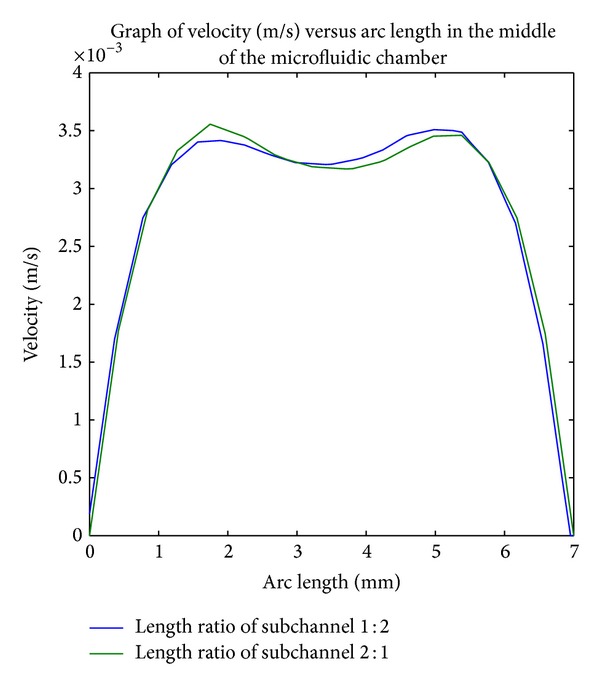
Graph of velocity versus arc length in the middle of microfluidic chamber (subchannel length ratio 1 : 2 & 2 : 1).

In general, the surface velocity plot and arrow plot for both architectures indicate a uniform distribution across the channel. However, in the process of further validating the result, an analysis about velocity fluctuation in the middle of both microfluidic channels is implemented. As COMSOL Multiphysics software merely provides individual design analysis, the velocity magnitude data are exported from COMSOL in order to compare the velocity flow within both architectures. The data are then plotted by using MATLAB R2010a software. The graph of average velocity field in the middle of the analysis area for subchannel with length ratio of 1 : 2 and 2 : 1 is delivered in Figure 6. To simplify the explanation, microfluidic with subchannel's length ratio of 1 : 2 is represented with Model A while microfluidic with subchannel's lengh ratio of 2 : 1 is Model B.

From the graph, the maximum velocity field achieved by both models is 3.5 mms^−1^. However, Model A has more stable uniform velocity flow across the channel compared to Model B. This condition which is caused by longer downstream subchannel compared to its upstream channel allows the higher resistance to be generated within it. As a result, the nonuniform distribution of particles among microfluidic channel that are established in first bifurcation channel can be removed through its longer downstream channel. Henceforth, it proves that Model A is able to develop a better flow distribution which flattens the velocity particle suspension from the inlet uniformly in contrast to Model B.

Although both models show higher hydraulic resistance than the original proposed microfluidic, they have higher mean velocity field in the middle of chamber. Such a condition can be explained with Chang et al. finding [33], where the increase of subchannel length will cause generation of Poiseuille flow within them over time. When the fluid flows from subchannel toward the chamber, the Poiseuille flow will cause the mean velocity field to be slightly higher compared to our original design. Since the fluid flow velocity across the chamber is fluctuated around 3.5 mms^−1^, it does not fulfill the requirement of the optimum value to allow sufficient cell capture within the chamber for DEP.

### 4.3. Effect of Bifurcation

**Figure 7:**
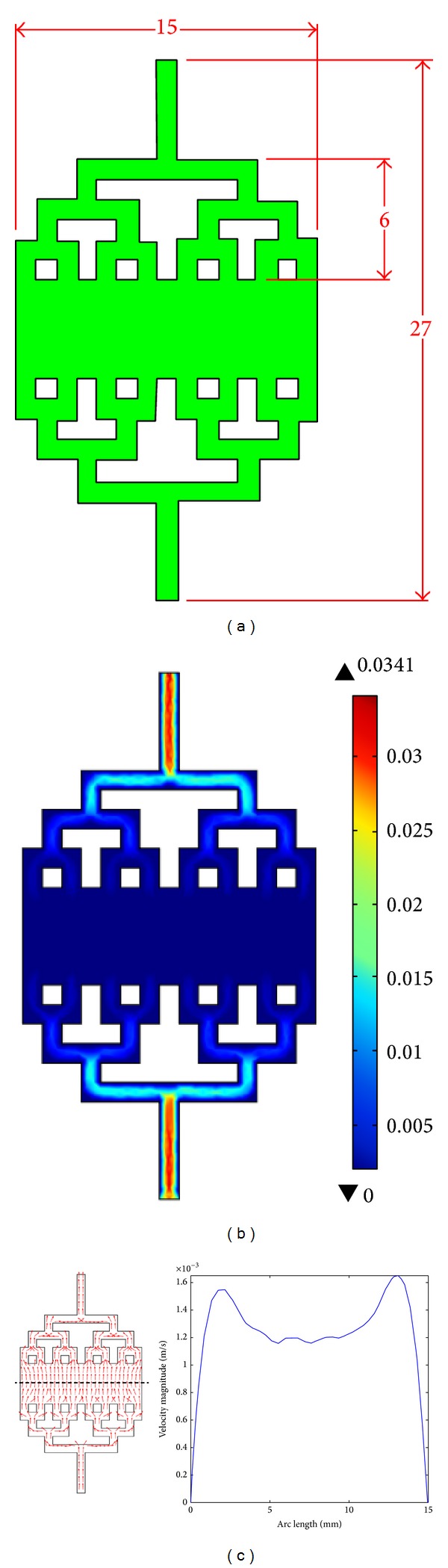
Schematic of (a) microfluidic channel with 3-level of bifurcation with its (b) surface velocity plot; (c) arrow plot and graph of velocity profile at the middle of microfluidic chamber (represented with dash line).

Apart from length fraction between subchannels, the effect of bifurcation on microfluidic channel geometry is investigated. Previous simulation has shown that the presence of bifurcation has developed portioning of volumetric flow which produces uniform velocity flow across the channel. In this section, a design of 3-level bifurcation microfluidic channel is investigated. The geometry of the microfluidic bifurcation is shown in Figure 7(a). Although both surface plot and arrow plot (see Figure 7(b)) show an uniform distribution of flow within the microfluidic channel, a comprehensive analysis of velocity profile in the middle of the chamber indicates that there is a slight drop of velocity magnitude in the centre of the middle cross-sectional area (see Figure 7(c)). The condition is particularly driven by the effect of electric double layer (EDL) around the channel surface which subsequently results in the pressure-driven flow on fluid near to the wall. Consequently, the fluid flow near the channel wall has higher velocity compared to the central region.

Besides, from Figure 7(c), the maximum velocity magnitude obtained in the middle of microfluidic channel is 0.0016, which halves the value of our original 2-level bifurcation proposed geometrical design. The matter of fact is that this condition is in agreement with Emerson et al. [31] study, which highlights that the volumetric flow rate is halved at each bifurcation. Even though this geometrical design allows the sufficient cell to be captured within the chamber because its maximum velocity magnitude is less than 0.003 ms^−1^, its value of velocity magnitude is the lowest compared to the previous design. Thus, the chance of deposition of cell suspension around the chamber is the highest particularly in the centre region, where DEP operation is implemented. Nonetheless, as opposed to our proposed design, the geometry is flaw because it will occupy a huge space in the microfluidic design. Eventually, there is a dire need to invest a large sum of money on a series of further experiments and great amount of reagent which is required to fill up the chamber. Ultimately, it is indeed compulsory for the researchers to carry out miniaturization to revise the design so that the geometry will appear to be more cost-effective and practical.

### 4.4. Design Improvement

**Figure 8:**
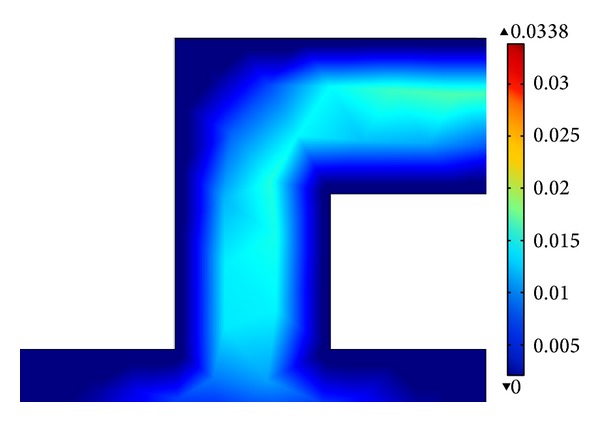
Schematic presentation of velocity flow at sharp corner of microfluidic channel.

Generally, the basic proposed microfluidic structure with 2-level of bifurcation and 1 : 1 subchannel length ratio appears to be the best performance to process a uniform velocity flow across the channel. In spite of the fact that a rectangular cross-sectional microfluidic channel is easy to fabricate, multiple zero velocity areas are normally produced in the corners. It is further evidenced through COMSOL simulation as presented in Figure 8, where the dark blue colour segment around the sharp corner represents a zero velocity profile. This situation can cause the large number of cells to be collected [34]. The lack of uniform adhesion in this region compared to others indicates that the channels with sharp turns are not optimal for DEP cell separation application.

**Figure 9:**
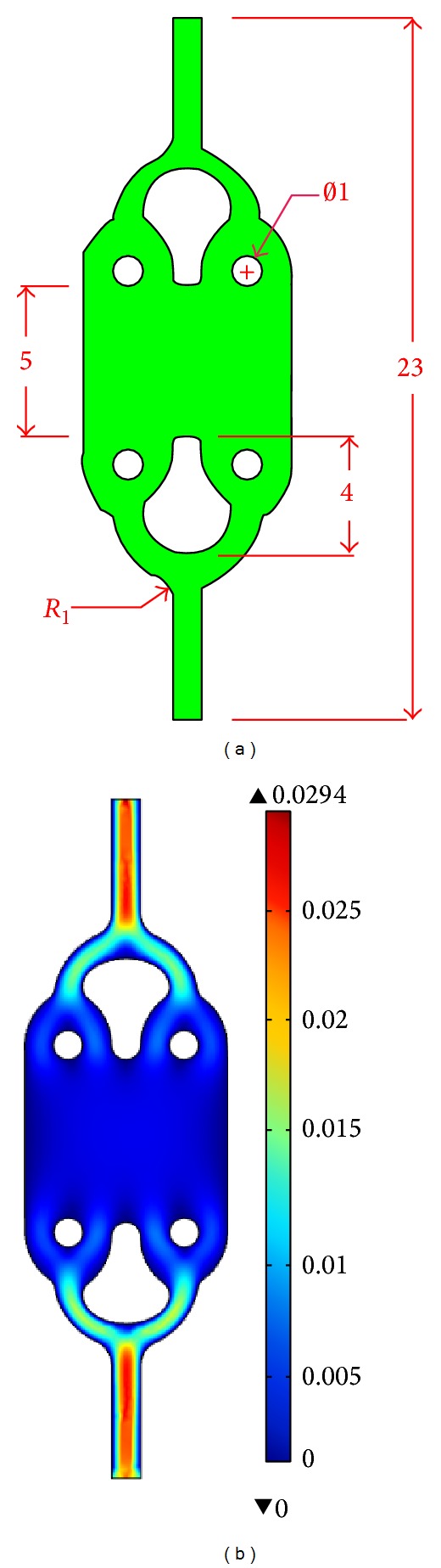
Schematic of (a) microfluidic channel with rounded corner and its (b) surface velocity plot.

By resorting to the study done by Feng et al. [30] and Green et al. [34], a round-shaped turn is identified to generate uniform velocity profiles and cell adhesion within the channel. Thus, the sequence of round-shaped turn is used to replace the sharp corner of our original proposed microfluidic channel architecture. The calculated compactness is 0.51 and its geometrical design is presented in Figure 9(a).

**Figure 10:**
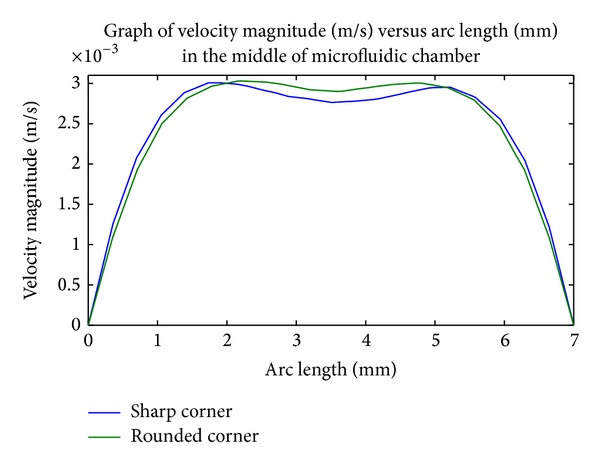
Comparison of average velocity across the width of microfluidic chamber for rounded corner and sharp corner.

In contrast to previous design in this study, the surface plot indicates a uniform velocity profile throughout the channel in the first bifurcation, which is represented with evenly cyan color segment (Figure 9(b)). Such a profile also implies that the shear stress within the channel is uniform, thereby predicting homogenous cell adhesion within it. Also, when velocity profiles for both original design (labeled with sharp corner) and improved design (labeled with rounded corner) are plotted with MATLAB, rounded corner provides the most uniform cell distribution across the width in the middle of microfluidic as shown in Figure 10.

Because of the improvement in velocity difference shown by this design, the rounded corner geometry is more preferable than our original proposed sharp turn design. Therefore, it is adopted as the new design to be used in DEP system.

## 5. Conclusion

Overall, the present work was set out with the objective to develop a new microfluidic channel geometry for the use of DEP application. The general strategy of achieving this aim is by first illustrating a set of design criteria for DEP application microfluidic channels such as optimum fluid mean velocity, uniformity of flow, compactness, and fabrication complexity. Using this strategy, the tree-like flow division network architecture was purposed to help generate a uniform distribution across the microfluidic channel. The presence of 2-level bifurcation in this architecture enables the nonuniformity particle profile to be removed, thus producing a uniform velocity within the chamber. The rectangular shape of channel cross section was considered in this design due to its ease of fabrication.

Apparently, based on the numerical simulation of Navier-Stokes equation developed with COMSOL Multiphysics software, our proposed network division architecture has shown a great improvement compared to typical benchtop-fabricated microfluidic channel design. The uniform velocity flow within such a geometrical design allows uniform deposition of cell within the channel, thus fulfilling the microfluidic requirement for DEP application. At the moment, the proposed microfluidic design for DEP application is solely based on computer fluid modelling analysis. As such, there is a need for experimental characterization to verify the design strategy and simulation.
